# Characterization of a highly stable zwitterionic hydrophilic interaction chromatography stationary phase based on hybrid organic–inorganic particles

**DOI:** 10.1002/jssc.202100859

**Published:** 2022-01-17

**Authors:** Thomas H. Walter, Bonnie A. Alden, Kenneth Berthelette, Jessica A. Field, Nicole L. Lawrence, Justin McLaughlin, Amit V. Patel

**Affiliations:** ^1^ Chemistry R&D Waters Corporation Milford Massachusetts USA

**Keywords:** column selectivity, hybrid particles, hydrophilic interaction chromatography, pH stability, zwitterionic stationary phases

## Abstract

We have characterized a sulfobetaine stationary phase based on 1.7 μm ethylene‐bridged hybrid organic–inorganic particles, which is intended for use in hydrophilic interaction chromatography. The efficiency of a column packed with this material was determined as a function of flow rate, demonstrating a minimum reduced plate height of 2.4. The batch‐to‐batch reproducibility was assessed using the separation of a mixture of acids, bases, and neutrals. We compared the retention and selectivity of the hybrid sulfobetaine stationary phase to that of several benchmark materials. The hybrid sulfobetaine material gave strong retention for polar neutrals and high selectivity for methyl groups, hydroxy groups, and configurational isomers. Large differences in cation and anion retention were observed among the columns. We characterized the acid and base stability of the hybrid sulfobetaine stationary phase, using accelerated tests at pH 1.3 and 11.0, both at 70°C. The results support a recommended pH range of 2–10. We also investigated the performance of columns packed with this material for metal‐sensitive analytes, comparing conventional stainless steel column hardware to hardware that incorporates hybrid surface technology to mitigate interactions with metal surfaces. Compared to the conventional columns, the hybrid surface technology columns showed a greatly improved peak shape.

**Article Related Abbreviations**
2dG: 2′‐deoxyguanosine
3dG: 3′‐deoxyguanosine
A: adenosine
AA: ammonium acetate
ADP: adenosine diphosphate
AF: ammonium formate
AmBic: ammonium bicarbonate
AMP: adenosine monophosphate
As_10_
: peak asymmetry at 10% peak height
ATP: adenosine triphosphate
BEH: ethylene‐bridged hybrid
dU: deoxyuridine
*h*_min_
: minimum reduced plate height
HST: hybrid surface technology
*k*
: retention factor
MeU: 5‐methyluridine
*r*
: relative retention
Tb: theobromine
TMPA: *N,N,N*‐trimethylphenylammonium chloride
Tp: theophylline
TS: sodium *p*‐toluenesulfonate
U: uridine
V: vidarabine
α: 4‐nitrophenyl α‐d‐glucopyranoside
β: 4‐nitrophenyl β‐d‐glucopyranoside

## 1 INTRODUCTION

Hydrophilic interaction chromatography (HILIC) is one of the most useful separation techniques for polar analytes because it has the desirable feature that retention increases with increasing analyte polarity [1, 2]. A variant of normal phase chromatography, HILIC involves the use of a polar stationary phase and a less polar organic–aqueous mobile phase [1]. A wide variety of HILIC stationary phases have been reported, including neutral, charged, and zwitterionic materials [3, 4]. Of the zwitterionic stationary phases, materials derivatized with sulfobetaine groups have been the most studied [5, 6, 7, 8, 9, 10, 11, 12]. Bearing sulfonate and quaternary ammonium groups in a 1:1 molar ratio, sulfobetaine groups are net neutral over the pH range of 0–14 and consequently exhibit relatively weak ion‐exchange behavior [13]. Sulfobetaine stationary phases have been shown to accumulate a relatively thick layer of adsorbed water [14, 15, 16, 17], which makes them strongly retentive for polar neutrals in HILIC. The combination of high retentivity and a net‐zero surface charge has made sulfobetaine columns a popular choice for a wide range of applications, including metabolomics [18], cell culture media analysis [19], pharmaceutical bioanalysis and impurity profiling [20, 21], determination of the concentrations of toxins in food [22, 23, 24], and analyses of biopolymers including peptides [25, 26], glycoproteins [26], and oligonucleotides [27].

Most of the commercially available sulfobetaine stationary phases are based on silica particles. Because of the dissolution of silica in basic solutions, these materials are only compatible with mobile phases having a pH below approximately 8. However, using mobile phases containing alkaline buffers (e.g., pH 9.2) has been shown to give the lowest limits of quantification and the widest linear dynamic ranges for LC–MS/MS analyses of 50 key hydrophilic cellular metabolites [28]. Other reports have also demonstrated that alkaline buffers (ca. pH 9) give better peak widths, peak shapes, and S/N ratios for organic acids and phosphates in polar metabolomics investigations [29, 30, 31]. These studies used an organic polymer‐based sulfobetaine stationary phase (Merck ZIC‐pHILIC) which is reported to be stable from pH 2–10 [32]. However, columns packed with this material are only available with 5 μm particles and have maximum efficiencies of only 40 000 plates/m. An alternative solution to impart high pH stability is the use of hybrid organic/inorganic particles. Ethylene‐bridged hybrid (BEH) particles have been shown to exhibit good stability in basic mobile phases [33, 34] as well as excellent mechanical strength, enabling their use in <2 μm particle sizes for UPLC [35]. Columns packed with 1.7 μm BEH HILIC particles have been reported to have maximum efficiencies of 227 000 plates/m [36].

A sulfobetaine stationary phase based on BEH particles was recently introduced. Here, we describe the characterization of this stationary phase for column efficiency, batch‐to‐batch reproducibility, and acid and base stability. The retention and selectivity of the hybrid sulfobetaine material were compared to those of two other HILIC stationary phases based on BEH particles: BEH Amide and BEH HILIC. For these comparisons, we used a previously reported set of test compounds that were chosen to assess the selectivity for hydrophilic and hydrophobic substituents, for regio‐ and configurational isomers, for molecular shape, for surface acidity, and for cations and anions [37]. Results have previously been reported for 45 different HILIC columns, making this a useful data set for comparisons [38]. In addition, we characterized the performance of the hybrid sulfobetaine material for three phosphorylated compounds, comparing the results obtained using conventional stainless steel column hardware to those observed using hardware modified with hybrid surface technology [39].

## 2 MATERIALS AND METHODS

### 2.1 Chemicals

LC–MS‐grade acetonitrile was obtained from Fisher Scientific (Hampton, NH, USA). Ammonium formate (AF), ammonium acetate (AA), ammonium bicarbonate (AmBic), trifluoroacetic acid (TFA), and all analytes were sourced from Millipore–Sigma (Burlington, MA, USA). Deionized water was produced using a Milli‐Q system (Millipore–Sigma, Burlington, MA, USA).

### 2.2 Instrumentation and columns

All chromatographic evaluations were performed using ACQUITY UPLC Classic, H‐Class, I‐Class, or Premier Systems equipped with ACQUITY photodiode array detectors (Waters Corporation, Milford, MA, USA). ACQUITY UPLC BEH Amide, BEH HILIC, and Atlantis Premier BEH Z‐HILIC columns (1.7 μm, 2.1 × 50 mm) were obtained from Waters (Milford, MA, USA).

### 2.3 Sample and mobile phase preparation

The pH values for all buffer solutions were determined as aqueous solutions, before combining with acetonitrile, and the pH meter was calibrated using aqueous reference buffers. The buffer concentrations indicated below are the concentrations in the aqueous portion of the mobile phase, before mixing with acetonitrile.

The sample used for the column efficiency test contained 120 μg/mL acenaphthene (the hold‐up time marker) and 80 μg/mL cytosine dissolved in 80/20, v/v, acetonitrile/100 mM AF pH 3.0 (aq). The reproducibility test mixture contained 19 μg/mL acenaphthene, 3.7 μg/mL thymine, 25 μg/mL phthalic acid, 3.7 μg/mL adenine, 7.7 μg/mL cytosine, and 25 μg/mL 5‐fluoroortic acid, dissolved in 90/10, v/v, acetonitrile/100 mM AF pH 3.00 (aq). Ten samples were used for the selectivity test, the first containing 333 μg/mL sodium *p*‐toluenesulfonate (TS), 333 μg/mL *N,N,N*‐trimethylphenylammonium chloride (TMPA), the second containing 33.3 μg/mL uridine and 1.0 mg/mL toluene (the hold‐up time marker), the third containing 100 μg/mL 3′‐deoxyguanosine, 33.3 μg/mL vidarabine, and 33.3 μg/mL 5‐methyluridine, the fourth containing 33.3 μg/mL uridine and 100 μg/mL 2′‐deoxyguanosine, the fifth containing 33.3 μg/mL adenosine, and 33.3 μg/mL 2′‐deoxyuridine, the sixth containing 50 μg/mL theobromine, the seventh containing 50 μg/mL theophylline, the eighth containing 200 μg/mL 4‐nitrophenyl α‐d‐glucopyranoside, the ninth containing 200 μg/mL 4‐nitrophenyl β‐d‐glucopyranoside, and the tenth containing 1.0 mg/mL toluene. The first two samples were dissolved in 90/10, v/v, acetonitrile/100 mM AA pH 4.7 (aq) and the other samples were dissolved in 90/10, v/v, acetonitrile/20 mM AA pH 4.7 (aq). The sample for the base stability test contained 25 μg/mL acenaphthene, 50 μg/mL cytosine, 25 μg/mL adenine, and 400 μg/mL TS dissolved in 80/20, v/v, acetonitrile/water. The sample for the acid stability test contained 50 μg/mL toluene, 50 μg/mL cytosine, 50 μg/mL uridine, and 400 μg/mL TS dissolved in 0.36% TFA in 80/20, v/v, acetonitrile/water. For the metal‐sensitive analyte evaluation, we used separate samples containing 50 μg/mL each of adenosine monophosphate disodium salt (AMP), adenosine diphosphate disodium salt hydrate (ADP), and adenosine triphosphate disodium salt hydrate (ATP) dissolved in 70/30, v/v, acetonitrile/20 mM AA pH 6.8 (aq).

### 2.4 Method details

The column efficiency measurements were carried out using an ACQUITY I‐Class system with a mobile phase of 80/20, v/v, acetonitrile/100 mM AF pH 3.00, a temperature of 30°C, and UV absorbance detection (254 nm). The sample contained acenaphthene as the hold‐up time (*t*_0_) marker and cytosine as the retained analyte. Four sigma efficiencies were determined for cytosine. Reduced plate heights were calculated by dividing the plate heights by the average particle size [40]. The interstitial linear velocities were obtained from the ratio of column length to *t*_0_. The reduced linear velocities were calculated by dividing these values by the average particle size and the diffusion coefficient of cytosine in the mobile phase, 2.14 × 10^−5^ cm^2^/s, estimated using the Scheibel modification of the Wilke‐Chang equation [41].

The reproducibility tests were carried out using 2.1 × 50 mm columns with a 90/10, v/v, acetonitrile/100 mM AF pH 3.00 (aq) mobile phase at 0.5 mL/min, an injection volume of 3.0 μL, a temperature of 30°C, and UV absorbance detection (254 nm). Columns packed with 17 different batches of 1.7 μm BEH Z‐HILIC were evaluated. For each column, four consecutive separations of the sample were carried out, and the results from the fourth injection were used to calculate the retention factors and relative retentions. For each batch, four columns were tested, and the average values were calculated for the retention factors and relative retentions.

The retention and selectivity test we used was reported by Ikegami et al. [37, 38]. Isocratic separations were carried out using a 90/10, v/v, acetonitrile/20 mM AA pH 4.7 (aq) mobile phase at 0.2 mL/min, an injection volume of 3.0 μL, a temperature of 30°C, and UV absorbance detection (254 nm). The hold‐up times measured using toluene with the same mobile phase were used to calculate the retention factors. Following Ikegami et al., the ionized analytes (TS and TMPA) were separated using a 90/10, v/v, acetonitrile/100 mM AA pH 4.7 (aq) mobile phase, and the TS/uridine and TMPA/uridine relative retentions were calculated using the uridine retention factors determined with the 90/10, v/v, acetonitrile/20 mM AA pH 4.7 (aq) mobile phase.

The accelerated base stability test was adapted from a previously described protocol [42]. Two 2.1 × 50 mm BEH Z‐HILIC columns were evaluated. The gradient program is shown in Table 1. One microliter of a sample containing acenaphthene, cytosine, adenine, and TS was separated using a 95/5, v/v, acetonitrile/100 mM AF pH 3.00 (aq) mobile phase at a flow rate of 0.4 mL/min with UV absorbance detection (254 nm). A challenge mobile phase containing 60/40, v/v, acetonitrile/10 mM AmBic pH 11.00 (aq) was then passed through the column at 0.4 mL/min for 20.57 min, followed by washing with 50/50, v/v, and 90/10, v/v, acetonitrile/water, then equilibrating with the 95/5, v/v, acetonitrile/100 mM AF pH 3.00 (aq) test mobile phase (all at 0.4 mL/min). This cycle was repeated 100 times, with the column temperature maintained at 70°C throughout the test. The efficiencies and retention factors of cytosine, adenine, and TS were determined versus the time exposed to the challenge mobile phase. A 5‐point boxcar average was used to increase the S/N ratio.

**TABLE 1:** Gradient program for the high pH stability test

**Time (min)**	**Flow rate (mL/min)**	**A (%) ‐ Water**	**B (%) ‐ ACN**	**C** * (%)	**D** ** (%)	**Curve**
initial	0.4	0	95	5	0	initial
13.73	0.4	0	0	0	100	11
34.30	0.4	0	0	0	100	11
35.97	0.4	50	50	0	0	6
39.27	0.4	50	50	0	0	6
40.93	0.4	10	90	0	0	6
44.23	0.4	10	90	0	0	6
45.90	0.4	0	95	5	0	6
68.13	0.4	0	95	5	0	11

* Mobile phase C was 100 mM AF pH 3 (aq).

** Mobile phase D was 60/40, v/v, ACN/10 mM AmBic pH 11 (aq).

The accelerated acid stability test was based on a protocol that was previously described [42]. Three 2.1 × 50 mm BEH Z‐HILIC columns were evaluated using a mobile phase gradient starting with a 0.25 min hold at 5% A, then a linear increase to 50% A over 2.75 min, a hold at 50% A for 0.5 min, a linear decrease back to 5% A over 0.5 min and a hold at that composition for 16 min. Mobile phase A was 0.5% TFA in water, mobile phase B was 0.5% TFA in acetonitrile, the flow rate was 0.4 mL/min, the temperature was 70°C, and UV absorbance detection (254 nm) was used. After equilibrating the columns for 10 min and making six blank injections, sixty‐one 1 μL injections of a sample containing toluene, cytosine, uridine, and TS were carried out. One of the three columns was tested for a longer time, with 128 injections being made. The retention times were determined as a function of the time exposed to the mobile phases. A 5‐point boxcar average was used to increase the S/N ratio.

The ATP/ADP/AMP tests were carried out using an ACQUITY Premier System with a mobile phase containing 70/30, v/v, acetonitrile/20 mM AA pH 6.8 (aq), a flow rate of 0.5 mL/min, a temperature of 30°C, and UV absorbance detection (260 nm). Separate samples of ATP, ADP, and AMP were used, with an injection volume of 0.4 μL. The injected mass was 20 ng for each nucleotide. The peak asymmetries at 10% peak height (As_10_) were determined, as well as the peak areas.

## 3 RESULTS AND DISCUSSION

### 3.1 Chemical and physical properties of the stationary phases

In Table 2, we detail the chemical and physical properties of the hybrid sulfobetaine (BEH Z‐HILIC) stationary phase as well as those of BEH Amide and BEH HILIC. The ethylene‐bridged hybrid particles used for the BEH Z‐HILIC stationary phase have an average pore diameter of 95 Å, smaller than the 130 Å particles used for the BEH Amide and BEH HILIC stationary phases. The 95 Å particles have a 46% higher surface area, which results in increased retention. The particles were derivatized with sulfobetaine groups, with an average surface concentration of 3.0 μmol/m^2^, calculated based on the nitrogen content and the surface area of the unbonded particles.

**TABLE 2:** Comparison of the chemical and physical properties of the stationary phases evaluated

	**Particle properties**			**Bonded phase properties**	
**Stationary Phase**	**Average pore diameter (Å)**	**Pore volume (cm^3^/g)**	**Surface area (m^2^/g)**	**Surface chemistry**	**Surface concentration (μmol/m^2^)**
BEH Z‐HILIC	95	0.7	270	sulfobetaine	3.0
BEH Amide	130	0.7	185	amide	7.5
BEH HILIC	130	0.7	185	unbonded	N.A.

### 3.2 Column efficiency

**FIGURE 1:**
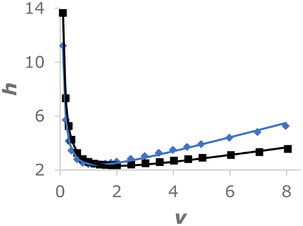
Reduced plate height (*h*) versus reduced linear velocity (*ν*) for 2.1 × 50 mm columns packed with 1.7 μm BEH Z‐HILIC or BEH Amide. The blue diamonds are the experimental values for the BEH Z‐HILIC column, and the blue line is the best fit to the reduced van Deemter equation (*h* = 0.90 + 0.99/*ν* + 0.559*ν*). The black squares are the experimental values for the BEH Amide column, and the black line is the best fit to the reduced van Deemter equation (*h* = 1.06 + 1.25/*ν* + 0.306*ν*). The retained compound was cytosine and the mobile phase was 80/20 v/v acetonitrile/100 mM AF pH 3.00 (aq)

To evaluate the efficiency of a 1.7 μm BEH Z‐HILIC 2.1 × 50 mm column, we carried out a van Deemter study. For comparison, a 1.7 μm BEH Amide 2.1 × 50 mm column was also tested. The results are shown in Figure 1 as plots of reduced plate height (*h*) versus reduced linear velocity (*ν*), along with the best fits to the reduced van Deemter equation. For the BEH Z‐HILIC column, a minimum reduced plate height (*h*_min_) of 2.43 was determined, slightly higher than that of the BEH Amide column (*h*_min_ = 2.34), but lower than the value of 2.59 previously reported for a 1.7 μm BEH HILIC column [36]. The *h*_min_ of 2.43 for the BEH Z‐HILIC column corresponds to a minimum plate height of 4.34 μm and a maximum efficiency of 230 000 plates/m. The optimum reduced linear velocity for the BEH Z‐HILIC column was 1.3, corresponding to a flow rate of 0.13 mL/min. This is lower than the optimum reduced linear velocity of 1.9 for the BEH Amide column and the optimum reduced velocity of 2.3 previously reported for a 1.7 μm BEH HILIC column [36]. The reasons for these differences are under investigation.

### 3.3 Batch‐to‐batch reproducibility

**FIGURE 2:**
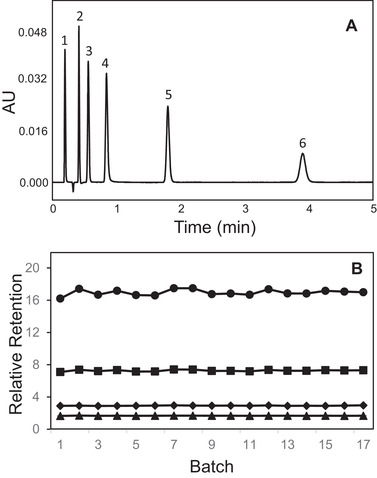
(A) Typical separation of the acids, bases, and neutrals mixture using a 2.1 × 50 mm 1.7 μm BEH Z‐HILIC column with a mobile phase of 90/10, v/v, acetonitrile/100 mM ammonium formate pH 3.0 (aq). Peak identification: 1: acenaphthene, 2: thymine, 3: phthalic acid, 4: adenine, 5: cytosine, 6: 5‐fluoroorotic acid. (B) Relative retentions for 17 different batches of 1.7 μm BEH Z‐HILIC for this separation. From top to bottom: *r*(5‐fluoroorotic acid/thymine), *r*(cytosine/thymine), *r*(adenine/thymine), *r*(phthalic acid/thymine)

The batch‐to‐batch reproducibility of the 1.7 μm BEH Z‐HILIC stationary phase was evaluated using an isocratic separation of five analytes, comprising acidic, basic, and neutral compounds. This sample mixture (without phthalic acid) and mobile phase were previously used to compare the selectivities of different HILIC stationary phases [43]. A representative separation is shown in Figure 2A. The relative retentions determined for 17 batches of the 1.7 μm BEH Z‐HILIC stationary phase are shown in Figure 2B. The results demonstrate good reproducibility, with relative standard deviations (RSD) ranging from 0.7% for *r*(phthalic acid/thymine) to 2.2% for *r*(5‐fluoroorotic acid/thymine). These RSD values are similar to those of the most reproducible C_18_ columns [44].

### 3.4 Retention and selectivity

To evaluate the retention and selectivity of the BEH Z‐HILIC stationary phase, we used tests described by Ikegami et al. [37, 38]. These tests employ a set of compounds chosen to probe different types of selectivity. The relative retention of uridine versus 5‐methyluridine is a measure of the methyl group selectivity, that of uridine versus 2′‐deoxyuridine is an indicator of the hydroxy group selectivity, *r*(vidarabine/adenosine) probes the configurational isomer selectivity, *r*(2′‐deoxyguanosine/ 3′‐deoxyguanosine) shows the regioisomer selectivity, *r*(4‐nitrophenyl α‐d‐glucopyranoside/4‐nitrophenyl β‐d‐glucopyranoside) indicates the shape selectivity*, r*(theobromine/theophylline) probes the surface acidity, *r*(trimethylphenylammonium chloride/uridine) indicates the cation‐exchange selectivity and *r*(sodium p‐toluenesulfonate/uridine) probes the anion‐exchange selectivity.

**FIGURE 3:**
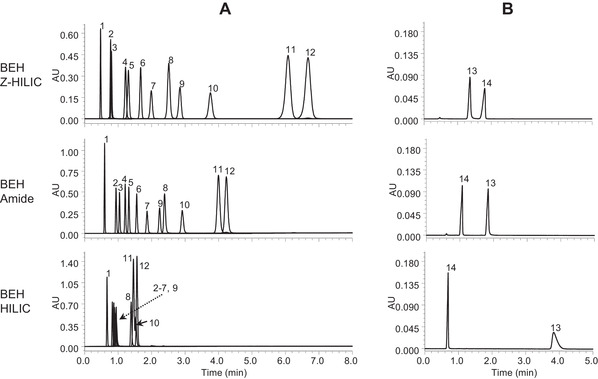
Isocratic separations of the Ikegami test compounds using BEH Z‐HILIC, BEH Amide, and BEH HILIC columns, all 1.7 μm 2.1 × 50 mm. Peak identification: 1: toluene (dead time marker), 2: theophylline, 3: theobromine, 4: 4‐nitrophenyl β‐D‐glucopyranoside, 5: 4‐nitrophenyl α‐D‐glucopyranoside 6: 2′‐deoxyuridine, 7: 5‐methyluridine, 8: adenosine, 9: uridine, 10: vidarabine, 11: 3′‐deoxyguanosine, 12: 2′‐deoxyguanosine, 13: *N,N,N*‐trimethylphenylammonium chloride, 14: sodium *p*‐toluenesulfonate; (A) the mobile phase was 90/10, v/v, acetonitrile/20 mM AA pH 4.7 (aq); (B) the mobile phase was 90/10, v/v, acetonitrile/100 mM AA pH 4.7 (aq)

Shown in Figure 3 are comparisons of the chromatograms obtained for these compounds using BEH Z‐HILIC, BEH Amide, and BEH HILIC columns. The results demonstrate that the BEH Z‐HILIC column gave the longest retention times for the majority of the compounds, while the BEH HILIC column gave the shortest. The retention factors and the relative retentions for the key analyte pairs are summarized in Table 3. The results show that, compared to the other stationary phases based on BEH, the BEH Z‐HILIC material had the highest values for *r*(uridine/5‐methyl uridine), *r*(uridine/2′‐deoxyuridine) (r(U/dU)), and *r*(vidarabine/adenosine). The lower *r*(4‐nitrophenyl α‐d‐glucopyranoside/4‐nitrophenyl β‐d‐glucopyranoside) (*r*(α/β)) value for the BEH Z‐HILIC stationary phase relative to BEH HILIC is consistent with the observation that the highest *r*(α/β) values were found for unbonded silica columns [37]. The relative retention of theobromine versus theophylline (*r*(Tb/Tp)) is believed to indicate the surface acidity, with higher values indicating greater acidity [37]. The lower *r*(Tb/Tp) value for the BEH Z‐HILIC column thus shows that this hybrid zwitterionic material is less acidic than the other BEH‐based stationary phases. The values of *r*(U/dU) and *r*(Tb/Tp) for the BEH Z‐HILIC stationary phase are consistent with the surface modification involving polymer grafting as opposed to simple silane bonding [38].

**TABLE 3:** Comparison of retention factors and relative retentions for five HILIC columns

**Parameter**	**BEH HILIC**	**BEH Amide**	**BEH Z‐HILIC**	**ZIC‐pHILIC** **	**ZIC‐cHILIC** **
*k* Theophylline	0.34	0.55	0.59	0.65	0.52
*k* Theobromine	0.39	0.73	0.64	0.71	0.47
*k* 4‐Nitrophenyl‐β‐d‐glucopyranoside	0.24	1.01	1.50	1.20	1.70
k 4‐Nitrophenyl‐α‐d‐glucopyranoside	0.30	1.19	1.68	1.38	1.93
*k* 2′‐Deoxyuridine	0.41	1.57	2.45	2.06	1.81
*k* 5‐Methyluridine	0.38	2.09	3.10	2.47	2.34
*k* Uridine	0.43	2.71	4.81	4.17	3.32
*k* Adenosine	1.08	2.94	4.19	3.49	2.75
*k* Vidarabine	1.29	3.82	6.71	4.94	4.54
*k* 3′‐Deoxyguanosine	1.18	5.60	11.43	8.89	7.52
*k* 2′‐Deoxyguanosine	1.34	6.00	12.69	9.89	8.41
*k p*‐Toluene sulfonate*	0.06	0.80	2.84	2.40	2.05
*k N,N,N*‐Trimethylphenylammonium*	4.69	2.08	1.92	1.19	1.41
*r*(U/MeU)	1.12	1.30	1.55	1.67	1.45
*r*(U/dU)	1.05	1.73	1.96	2.01	1.87
*r*(V/A)	1.20	1.30	1.60	1.41	1.65
*r*(α/β)	1.26	1.18	1.12	1.16	1.13
*r*(Tb/Tp)	1.16	1.31	1.09	1.09	0.91
*r*(2dG/3dG)	1.13	1.07	1.11	1.11	1.12
*r*(TS/U)*	0.14	0.30	0.59	0.58	0.62
*r*(TMPA/U)*	10.9	0.77	0.40	0.28	0.42
Euclidean distance versus BEH Z‐HILIC	10.6	0.70		0.26	0.24

* Values obtained using a mobile phase buffer concentration of 10 mM, all others used 2 mM.

** Values from reference 38.

The retention times for the two ionized compounds, TMPA and TS, show the largest differences between the BEH columns, as illustrated in Figure 3B. The BEH Z‐HILIC column gave greater retention for the anion TS than did the BEH Amide and BEH HILIC columns, while the opposite is true for the cation TMPA. This indicates that the BEH Z‐HILIC stationary phase has a less negative surface charge than the BEH Amide and unbonded BEH materials with this mobile phase, which is consistent with the conclusion from the *r*(Tb/Tp) results that the BEH Z‐HILIC material is less acidic. The BEH HILIC stationary phase shows the greatest retention of TMPA, consistent with a negative surface charge due to the ionization of surface silanols on the unbonded BEH particles. The differences in selectivity between these three stationary phases make them a useful set to screen during method development.

To compare the relative retentions for the BEH Z‐HILIC column to those previously reported for other HILIC columns, we calculated the Euclidean distances between the results for the BEH Z‐HILIC column and those for the 28 columns for which all eight relative retentions were given [37, 38]. The shortest distances were found for the ZIC‐cHILIC and ZIC‐pHILIC columns, indicating that the BEH Z‐HILIC column has the greatest similarity to these columns. The previously reported retention factors and relative retentions for the ZIC‐cHILIC and ZIC‐pHILIC columns are shown in Table 3. The ZIC‐pHILIC stationary phase has sulfobetaine groups attached to organic polymer particles, while the ZIC‐cHILIC stationary has phosphorylcholine groups attached to silica particles. Except for the TMPA/uridine relative retention, the *r* values for the BEH Z‐HILIC and ZIC‐pHILIC columns are within 12.2%. The larger difference (35%) in the TMPA/uridine relative retention may be due to the different particle substrates. The BEH particles have silanols on their surface, some of which may be ionized under the separation conditions, and thus capable of retaining cations like TMPA. In contrast, the organic polymer particles used for ZIC‐pHILIC lack these groups. Comparing the relative retentions of the BEH Z‐HILIC and ZIC‐cHILIC columns, the differences for the neutral analytes are slightly larger than for the BEH Z‐HILIC/ZIC‐pHILIC comparison (up to 17.5%), but the TMPA/uridine relative retentions are more similar (5% difference). This is consistent with a significant contribution from ionized silanol groups to the retention of TMPA.

### 3.5 Hydrolytic stability

**FIGURE 4:**
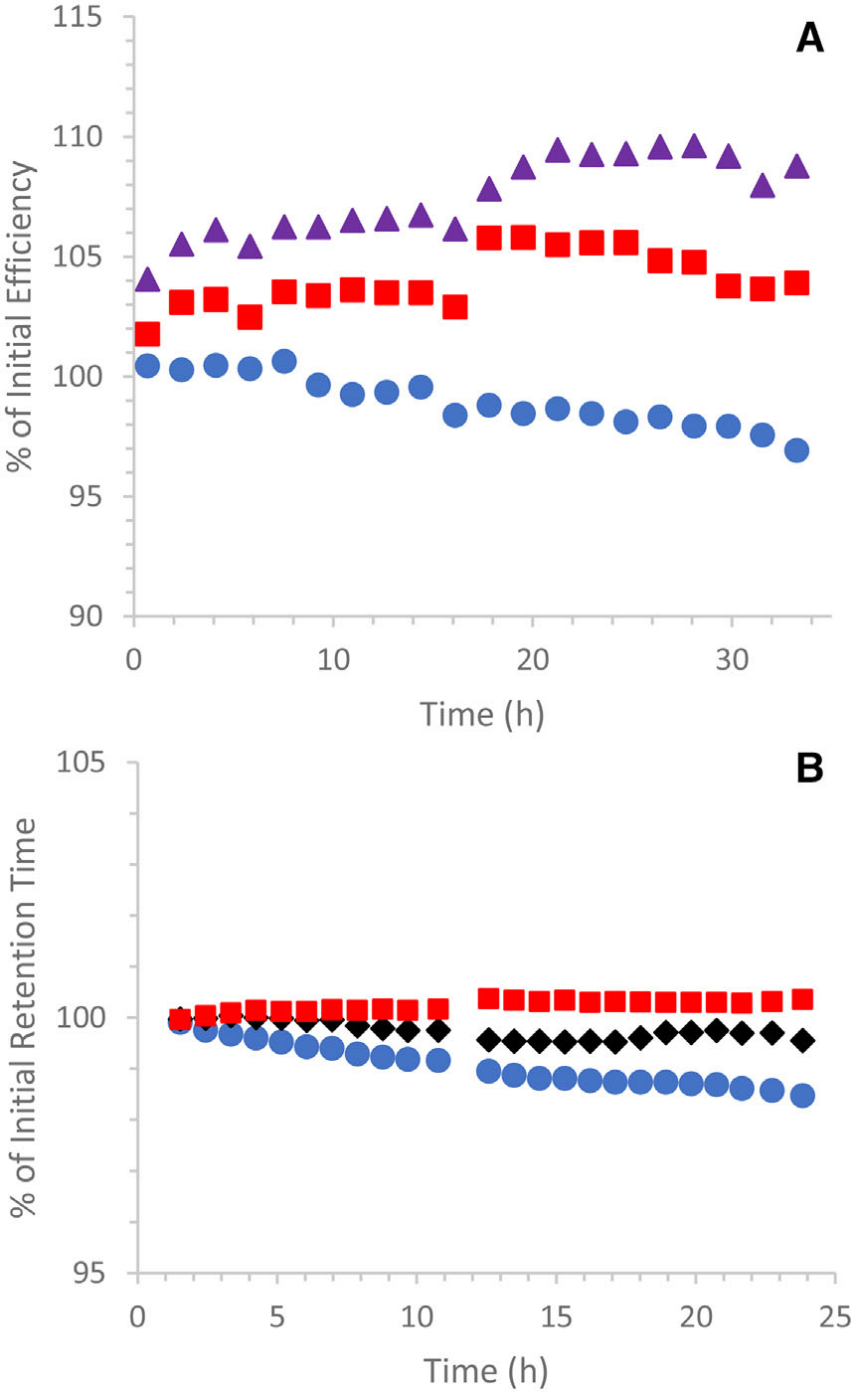
Accelerated stability test results for 2.1 × 50 mm 1.7 μm BEH Z‐HILIC columns; (A) The column was exposed to 60/40, v/v, acetonitrile/10 mM AmBic pH 11 (aq) at 70°C for 20.6 min during each injection cycle and the condition of the column was monitored using the separation of acenaphthene, adenine, cytosine, and TS with a mobile phase of 95/5 v/v acetonitrile/100 mM AF pH 3 (aq). The efficiencies as a percentage of the initial values are shown versus the time exposed to the pH 11 mobile phase (purple triangles, adenine; red squares, TS; blue circles, cytosine). (B) A mixture of toluene, cytosine, uridine, and TS were separated using a gradient with acetonitrile and water mobile phases containing 0.5% TFA (pH 1.2) and a temperature of 70°C. The retention times as a percentage of the initial values are shown versus the time exposed to the pH 1.2 mobile phases (black diamonds, uridine; red squares, TS; blue circles, cytosine)

To evaluate the acid and base stability of the hybrid sulfobetaine columns, we carried out accelerated tests at extreme pH values and elevated temperature. The base stability evaluation employed a challenge mobile phase containing 60/40, v/v, acetonitrile/10 mM AmBic (pH 11.00) and a temperature of 70°C. The column efficiency and retention were characterized by separating a mixture containing acenaphthene (the hold‐up time marker), adenine, cytosine, and TS using a 95/5, v/v, acetonitrile/100 mM AF (pH 3.00) mobile phase. Under the separation conditions, adenine and cytosine are positively charged while TS is negatively charged. After exposure to the challenge mobile phase for 20.57 min, the column was washed with 50/50, v/v, acetonitrile/water for 3.30 min, then 90/10, v/v, acetonitrile/water for 3.30 min before equilibrating with the efficiency/retention test mobile phase for 22.23 min. This test‐challenge‐wash‐equilibrate cycle was repeated 100 times. HILIC columns packed with silica‐based stationary phases generally showed large (30–70%) efficiency losses in this test, due to hydrolysis of the silica particles caused by the pH 11 mobile phase [42]. However, columns packed with BEH‐based stationary phases were more stable under these conditions [42]. The dependence of efficiency on the time exposed to the pH 11 mobile phase for a 1.7 μm hybrid sulfobetaine column is shown in Figure 4A. After 34 h of exposure, the column exhibited a 10% increase in efficiency for adenine, a 6% increase for TS, and a 2% decrease for cytosine. A second column showed efficiency changes of +14, +14, and −1% for adenine, TS, and cytosine, respectively. Both columns exhibited small increases in the retention factors for all three compounds after 34 h of exposure, with 9.8 and 9.7% increases for adenine, 5.3 and 5.7% increases for cytosine, and 0 and 1.4% increases for TS. The pressures changed <2% for both columns after 34 h of exposure. Based on these results, the recommended upper limit for the hybrid sulfobetaine columns is pH 10.

The acid stability of the hybrid sulfobetaine columns was assessed using a gradient separation with 0.5% TFA (pH 1.2) present in both the acetonitrile and aqueous mobile phases and a temperature of 70°C. The condition of the column was monitored by separating a mixture of toluene, cytosine, uridine, and TS. Under the separation conditions, cytosine has a positive charge, TS is negatively charged and uridine is uncharged. Exposure to acidic mobile phases can result in hydrolysis of bonded phases, which causes changes in retention and selectivity [42]. After 22 h of exposure, the retention times changed less than 2% (see Figure 4B). Similar results were obtained for three columns, although two of them were only monitored for 11 h. Based on these results, the recommended lower limit for the hybrid sulfobetaine columns is pH 2, which is conservative given the small changes observed at pH 1.2.

### 3.6 Performance for metal‐sensitive analytes

**FIGURE 5:**
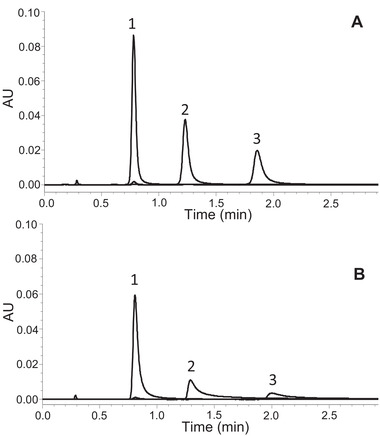
Separations of AMP, ADP, and ATP using 2.1 × 50 mm columns packed with 1.7 μm BEH Z‐HILIC in HST (A) or conventional stainless steel column hardware (B). Peak identification: 1: AMP, 2: ADP, 3: ATP. The mobile phase was 70/30, v/v, acetonitrile/20 mM AA pH 6.8 (aq)

Many polar compounds, particularly those that contain multiple carboxylate and/or phosphate groups, can interact with the stainless steel surfaces in HPLC columns [45, 46]. The effects of these interactions range from peak broadening and tailing to complete loss of analyte signal. To mitigate these effects, the column hardware for the hybrid sulfobetaine material has been modified using hybrid organic–inorganic surface technology (HST) [39]. An example of the improvement afforded by this column hardware is shown in Figure 5. Adenosine monophosphate (AMP), adenosine diphosphate (ADP), and adenosine triphosphate (ATP) were analyzed using an HST column and a conventional stainless steel column, both packed with the BEH Z‐HILIC stationary phase. With the conventional hardware, ADP and ATP gave broad, tailing peaks. However, when using the HST column all three nucleotides were observed as more symmetric peaks having the expected areas. The peak asymmetries (As_10_) and areas determined using the two different columns are compared in Table 4. This evaluation was carried out using an ACQUITY Premier System, in which HST has been used to mitigate adsorption in the UPLC instrument [47].

**TABLE 4:** Peak areas and asymmetries for conventional stainless steel versus hybrid surface technology columns packed with 1.7 μm BEH Z‐HILIC

**Column type**	**Analyte**	**As_10_**	**Peak area**
Conventional stainless steel	AMP	2.8	2.14 × 10^5^
	ADP	7.4	1.09 × 10^5^
	ATP	10.7	5.8 × 10^4^
Hybrid surface technology	AMP	1.5	2.24 × 10^5^
	ADP	1.6	1.49 × 10^5^
	ATP	1.8	1.15 × 10^5^

## 4 CONCLUDING REMARKS

These results demonstrate that columns packed with the 1.7 μm hybrid sulfobetaine material exhibited high efficiencies, good batch‐to‐batch reproducibility, and stability from pH 2 to 10. The BEH Z‐HILIC stationary phase gave strong retention for neutral polar analytes, greater than that of BEH Amide and BEH HILIC. The selectivity of the BEH Z‐HILIC material differs from that of the other two BEH‐based stationary phases, particularly for ionized compounds, with cations being less retained while anions are more retained. Based on a comparison to the relative retention results previously reported for 28 different HILIC columns, the hybrid sulfobetaine column was found to be most similar to ZIC‐pHILIC and ZIC‐cHILIC columns. Because of the use of hybrid surface technology for the column hardware, BEH Z‐HILIC columns enable high recoveries and good peak shapes for compounds that interact with metal surfaces. These attributes make BEH Z‐HILIC columns useful for a range of applications. The fit for polar metabolomics is particularly compelling, because of the common use of high pH (ca. 9) mobile phases and the need to achieve good peak shapes and high sensitivity for metal‐sensitive analytes such as organic acids, nucleotides, and other phosphorylated metabolites. Work is in progress to evaluate the use of these new columns for metabolomics applications [48].

## CONFLICT OF INTEREST

Except for Amit Patel, the authors are currently employed by Waters Corporation, the manufacturer of the columns that were evaluated.

## Data Availability

The data that support the findings of this study are available from the corresponding author upon reasonable request.
